# Analysis of Student Perceptions of Just-In-Time Teaching Pedagogy in PharmD Microbiology and Immunology Courses

**DOI:** 10.3389/fimmu.2020.00351

**Published:** 2020-02-28

**Authors:** Charitha Madiraju, Eglis Tellez-Corrales, Henry Hua, Jozef Stec, Andromeda M. Nauli, Deborah M. Brown

**Affiliations:** ^1^Department of Pharmaceutical Sciences, Marshall B. Ketchum University, Fullerton, CA, United States; ^2^Department of Pharmacy Practice, College of Pharmacy, Marshall B. Ketchum University, Fullerton, CA, United States; ^3^Nebraska Center for Virology, University of Nebraska - Lincoln, Lincoln, NE, United States

**Keywords:** just-in-time teaching (JiTT), Integrated Microbiology & Virology, Integrated Immunology, PharmD curriculum, instructional pedagogy

## Abstract

Just-In-Time Teaching (JiTT) active learning pedagogy is utilized by various disciplines, but its value in a professional pharmacy curriculum has not yet been demonstrated. The purpose of our research study is to implement and evaluate JiTT in a Doctor of Pharmacy (PharmD) program. The impetus in implementing JiTT into a PharmD curriculum was to provide students with an out-of-classroom learning opportunity to enhance knowledge-based skills. The current study summarizes the implementation of JiTT in four distinct instances: two iterations of the required courses “Integrated Microbiology and Virology” (Fall 2016 and Fall 2017) and “Integrated Immunology” (Winter 2016–2017 and Winter 2017–2018). JiTT included knowledge-based questions in multiple-choice format, integrated case studies, and student responses prior to the actual lecture session. After the conclusion of each course, students were asked to provide feedback on the utilization of JiTT by way of an anonymous survey. Following the Fall 2016 iteration of the Microbiology & Virology course, students found the integrated case studies to be beneficial (mean = 3.27 out of a maximum of 4, *SD* = 0.62), and their overall endorsement of JiTT was high (mean = 3.61 out of 4, *SD* = 0.50). For the other three courses included in this study, the primary dependent variable was the student's average rating of JiTT, rated on a five-point scale. Aggregating the scores from the Fall 2017 iteration of the Integrated Microbiology & Virology course and both instances of the Immunology course, students rated JiTT very favorably (mean = 4.17 out of a maximum of 5, *SD* = 0.77). Students' performances in JiTT-based courses were compared against non-JiTT-based courses. Analysis of assessment data for student's performance on knowledge-based questions showed JiTT was helpful for student learning and JiTT-based courses had more consistent exam scores compared to non-JiTT-based courses. The current results are a promising initial step in validating the usefulness of JiTT in a pharmacy program and lays the foundation for future studies aimed at a direct comparison between a traditional lecture style and JiTT pedagogy implemented into PharmD curricula.

## Introduction

Just-in-Time Teaching (JITT) is an active learning pedagogy aimed toward improving student learning skills and educational outcomes (1). JiTT technique essentially involves a feedback loop between the outside-of-class learning environments and the face-to-face classroom sessions (1). JiTT active learning strategy provides students with an opportunity to self-reflect on their level of understanding of the lecture material and on the prior knowledge they have on each lecture topic. The basis of JiTT active learning strategy requires students to work on individual assignments often referred to as “warm-ups” (2).

In JiTT technique, students are provided with an opportunity to work on an assignment (or assignments), based upon an upcoming lecture topic, before coming to an actual class session (1). Before each lecture session, the course instructor gathers student responses to the assignment, and obtains a fairly good impression of the following: (1) student's foundational knowledge relevant to the required reading material for the upcoming class, (2) concepts within the assigned reading material for the upcoming lecture topic that students find them are new and challenging, and (3) student's perception of the course material and subject matter. Student responses to a given JiTT assignment provide an opportunity for faculty to tailor the classroom lecture session “just-in-time” (1). Classroom session can then be utilized effectively to discuss JiTT assignments, address misconceptions, and troubleshoot a problem within a case study while discussing course content (3).

The usefulness of JiTT has been demonstrated across various disciplines (4). Results from assessment of JiTT approach implemented for biomechanics education indicated significantly higher learning gains and better understanding of a concept-based JiTT course, relative to a non-JiTT course (5). JiTT methodology effectively enhanced knowledge-based skills required for comprehensive understanding of topics including core health-care professional curricula (2, 6–10). Medical residency programs identified JiTT as an effective approach that helped residents in their interactive learning of clinical modules, increased learner participation during core sessions in the curriculum and enhanced retention of JiTT course content (7, 8). More recently, JiTT using video-based lectures (VBLs) was incorporated and was very well-perceived by students enrolled into a neurology clerkship program (9). Besides, it was successfully incorporated into neuroeducation study as a reinforcement-based learning tool to help establish the foundational knowledge of neuroanatomy in novice learners (10).

Analogous to JiTT, just-in-time (JiT) training strategy is a simulation-based training (11, 12). JiT training undertaken at a Pediatric Emergency Department was found to significantly improve medical students' and resident trainees' procedural skills, procedure-related knowledge, and comfort level of trainees to perform a given procedure (11, 12). Similarly, JiT training strategy was found to markedly improve knowledge of nursing training staff that brought prior JiT training information to the bedside educational discussions (13). JiT training tool was used to validate minimum competency of bedside nursing staff managing high-risk low-volume therapies in order to ensure patient safety (14). A recent literature report also suggested that JiT active learning of evidence-based healthcare curricula created an opportunity for students to engage with facilitators and peers, enhance knowledge-based skills, and increase their chances of reinforcing and retaining their curricular knowledge (15). It is established that active learning teaching practice benefits small class sizes to a greater extent while showing an overall gain in student performance in undergraduate science, technology, engineering, and mathematics (STEM) courses compared to a traditional lecturing approach (16). Active learning fosters opportunities for students to come prepared, stay engaged and develop specific process skills that help in integrating knowledge during their learning of the material (17, 18).

JiTT as an active learning tool was implemented previously in an upper-level undergraduate Immunology course (19, 20). Results from students' survey analysis indicated JiTT to have a positive impact on student learning of the Immunology course material. JiTT pedagogy was well-received by students enrolled into Immunology course and students perceived JiTT to be especially beneficial during problem-solving of the case studies (19, 20). The latter is very important because when it comes to health care professional courses like Immunology or Infectious Diseases, it is easier for students to recall basic science concepts as applicable to problem scenarios or clinical cases (21, 22). Hence, a sound knowledge of basic science concepts and recalling of the concepts is essential to initiate a thought-provoking discussion and problem-solving of clinical case studies; in this regard, JiTT pedagogical approach implemented for undergraduate Immunology course has been perceived to be beneficial (19, 20). Learner-centered active pedagogy and flipped classroom model approaches, implemented into integrated basic science curricular framework, were shown to not only facilitate student engagement during in-class discussion but also help with their understanding, retention and application of basic science curricular concepts (23, 24).

Unlike medical education programs, JiTT was not implemented into any Doctor of Pharmacy (PharmD) curricula. Depending on curricular innovation needs, several other active learning techniques have been implemented into Pharmacy curriculum, including: audience response system, interactive web-based learning, visual aids-based learning, team-based learning (TBL), problem-based learning (PBL), process-oriented guided inquiry learning (POGIL), patient simulation and also blended approach of embedding active learning instructional tools within traditional lectures (25–29). Based on these reports it is widely accepted that in pharmacy health professions field, compared to traditional instructor-centered teaching approaches, student-centered active learning pedagogies serve as essential tools that help students understand and apply core conceptual knowledge to clinical practice. There is a report on JiT training strategy incorporated into a simulated influenza vaccination clinic that had an objective to train student pharmacists in just-in-time format (compared to traditional training approach) for administering emergency pediatric influenza vaccine (30). This training of student pharmacists in a simulated influenza vaccine clinic elicited significantly positive outcomes in students, including: competency, confidence and comfort to administer emergency pediatric influenza vaccine (30).

The purpose of our research study is to implement and evaluate JiTT in a Doctor of Pharmacy (PharmD) educational program (31, 32). JiTT was developed and implemented for P1-PharmD Class 2020 and P1-PharmD Class 2021 Integrated Microbiology & Virology and Integrated Immunology courses offered during Fall and Winter quarters (31, 32). A survey was administered at the end of each quarter that provided an opportunity for students to assess their perceptions of JiTT. A comparison was made between students' performances on knowledge-based exam questions in JiTT- vs. non-JiTT- based courses in order to assess the helpfulness of JiTT.

The overarching goal of implementing JiTT into PharmD curriculum is to provide graduates with the best possible knowledge during the course of the curriculum. The hypothesis is that JiTT pedagogy is beneficial to the active learning of PharmD course material. The primary objective of implementing JiTT is to structure out-of-class time and equip students with the best possible resources that help students develop effective study skills during their learning careers (1). Toward this end, research questions included: (1) How did students perceive JiTT pedagogy implemented for Integrated Microbiology & Virology and Integrated Immunology courses? (2) Was JiTT pedagogy beneficial to student learning of the course material? (3) Was there any difference in student learning outcomes in JiTT-based courses compared to non-JiTT courses?

## Materials and Methods

### JiTT Pedagogy

JiTT education sessions consisted of assignments including required reading material (self-directed slide presentations), multiple-choice questions, and integrated case studies that were developed as part of the active learning teaching pedagogy. There were an average of 20 multiple-choice questions included into each JiTT assignment and an average of two case studies per topic. Prior to each class meeting, students were asked to read the required course material posted to the course website (Moodle) and complete out-of-classroom assignments including the aforementioned multiple-choice questions and/or integrated case study assessments. Integrated clinical case study assignments relevant to each lecture topic were designed and administered in group-based assessment format to improve learner participation. Students were assessed for competency with just-in-time learning skills through various forms of assessment (pertinent to JiTT assignments and required reading material) including daily graded in-class individual quizzes, graded in-class exams, problem-solving of case studies, and participation during lecture sessions. The instructor used students' responses to tailor each class session to clarify difficult concepts and address any misconceptions on a given topic during the class time. In-class active learning group exercises and discussion of integrated case studies further reinforced concepts outlined in JiTT assignments. JiTT was applied in two courses for multiple cohorts of students at Marshall B. Ketchum University College of Pharmacy. The first course was “Integrated Microbiology & Virology,” which is offered during the Fall quarter of the first year of pharmacy school. The second course was “Integrated Immunology,” which is offered during the Winter quarter of the first year of professional pharmacy curriculum.

### JiTT Implementation Approach

**Figure 1:**
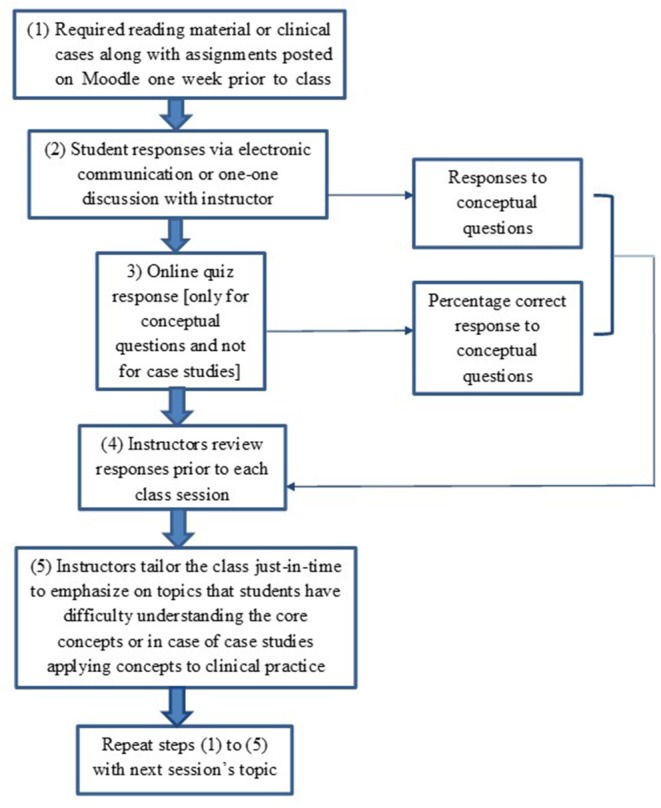
Flow Chart of just-in-time teaching pedagogy. Flow chart of just-in-time teaching pedagogy implemented for PharmD Microbiology and Immunology Courses.

The intention of JiTT is to provide an opportunity for students to participate in an out-of-classroom learning environment. Therefore, JiTT assignments pertaining to a given class session were posted on Moodle a week prior to that particular class session. Students are encouraged to ask questions to the course instructor and discuss with their peers, and are provided the opportunity to utilize office hours, electronic communication and engage in a discussion on topics that are difficult to comprehend. Course instructors note down student's responses prior to each class session. JiTT assignments prepared students for a closed-book quiz on ExamSoft prior to the class session. Students are not allowed to use their notes or assignment readings when taking the quiz. Instructors check the quiz performance and make a note of the percent response for each question, make notes on topic areas where students are having difficulty, and merge them with student responses obtained during out-of-classroom learning format such as one-one discussion or electronic communication with instructors. Instructors tailor their classroom environment to emphasize topic areas where students had difficulty. A flow chart depicting JiTT implementation approach for PharmD Microbiology and Immunology courses is shown in Figure 1. Various approaches including schematic models and flow charts are utilized to reiterate concepts from JiTT assignments that students identified as crucial gaps or missing links in their learning of key concepts.

Even though integrated case studies were developed separately, implementation procedure for case studies was similar to course material pertaining to conceptual knowledge (Figure 1). Case studies were implemented by correlating basic science concepts underlying Microbiology and Immunology with clinical information. Topics were selected depending on the relevance and frequency at which students encounter infectious diseases or immunological disorders in clinical practice. Some case studies were developed by the course instructors and some obtained from required textbooks or relevant literature. Cases were dispersed throughout the course and were posted on Moodle the week before class to provide an opportunity for students to participate in an out-of-classroom learning environment. Integrated case study assignments required students to work with their assigned team members. Students were encouraged to share their responses and ask questions to the course instructors during out-of-classroom learning prior to guided classroom discussions. Students analyzed the cases in team-based format and shared their responses either *via* one-to-one discussion with the instructor or electronic communication. Faculty made amendments in a JiTT format to tailor the class session to case studies where students had hard time applying their knowledge-based skills to clinical practice. Students had an opportunity to summarize the answers to the case studies in team-based format during in-class session in order to ensure correct understanding of the case studies.

JiTT consisted of assignments that included multiple-choice questions, integrated case studies and feedback obtained from students prior to actual lecture session. JiTT assignments were designed first to help students build a thorough understanding of the conceptual knowledge pertinent to Microbiology, Virology, and Immunology. Integrated case studies pertaining to a lecture topic were administered in JiTT format to help students translate basic conceptual knowledge into pharmacy practice. Students worked on JiTT assignments that encompassed conceptual knowledge related to the upcoming lectures and/or integrated case studies pertinent to the lecture topics, before coming to class sessions. At the beginning of the lecture sessions, course instructors tailored the respective session content to students' learning needs based on information gathered “just-in-time” from student responses to the individual assignments and topics that students had difficulty understanding. A survey was administered at the end of each quarter that provided an opportunity for students to assess their perceptions of JiTT. Participants of the survey included students form the two cohorts, PharmD Class 2020 and PharmD Class 2021 (see Table 1). Comparison was made between students' performances on knowledge-based exam questions in JiTT- vs. non-JiTT- based courses in order to assess the helpfulness of JiTT. Table 2 has the list of JiTT and non-JiTT courses offered to PharmD Class 2020 and PharmD Class 2021 cohorts.

**Table 1:** Student demographics.

**PharmD class year**	**Academic quarter**	**Gender**		**Ethnicity**					**BS/BA degree**	**Total**
		**Female**	**Male**	**Asian**	**Black**	**White**	**Hispanic**	**2 or more**		
2020	Fall 2016	26	17	27	4	10	1	1	37	43
	Winter 2016–2017	25	17	26	4	10	1	1	37	42
	Spring 2017	25	16	26	4	9	1	1	37	41
	Winter 2018–2019	23	15	24	4	8	1	1	37	38
2021	Fall 2017	34	22	33	3	15	2	3	52	56
	Winter 2017–2018	31	21	32	3	12	2	3	51	52
	Spring 2018	31	21	32	3	12	2	3	51	52

*Student demographics for PharmD Classes 2020 and 2021. Gender and ethnicity of students enrolled into each academic quarter are shown above. The academic degrees of students before joining PharmD program and the total number of students enrolled in each academic quarter are also shown. Demographic information shows a diverse group of students from PharmD Classes of 2020 and 2021 that participated in the survey*.

**Table 2:** List of JiTT and Non-JiTT courses.

**PharmD class cohort (year)**	**Academic quarter**	**JiTT courses**	**Non-JiTT courses**
2020		Course Title	Course Title
	Fall 2016 P1 Year	Integrated Microbiology & Virology	Pharmaceutical Biochemistry
			Foundations of Human Body & Disease – I
	Winter 2016–2017 P1 Year	Integrated Immunology	Foundations of Human Body & Disease – II
	Spring 2017 P1 Year	-	Foundations of Human Body & Disease – III
	Winter 2018–2019 P3 Year	Biotechnology, Pharmacogenomics & Precision Medicine	Biotechnology, Pharmacogenomics & Precision Medicine
2021	Fall 2017 P1 Year	Integrated Microbiology & Virology	Pharmaceutical Biochemistry
			Foundations of Human Body & Disease – I
	Winter 2017–2018 P1 Year	Integrated Immunology	Foundations of Human Body & Disease – II
	Spring 2018 P1 Year	-	Foundations of Human Body & Disease – III

*List of JiTT and non-JiTT courses utilized for comparison of student's performance in knowledge-based questions derived from individual assessments*.

### Study Participants

Demographic information of the study participants from the two cohorts (PharmD Class 2020 and PharmD Class 2021) is included in Table 1. The first cohort to take the JiTT and non-JiTT courses was the PharmD Class of 2020 (see Table 2 for the list of JiTT and non-JiTT courses offered for PharmD Class 2020 cohort of students). This cohort took a survey to rate their perceptions of JiTT after their Integrated Microbiology & Virology class concluded in Fall 2016, and all students enrolled in the class (*n* = 43) took the survey. This cohort then filled out a survey about their perception of JiTT after their Integrated Immunology course concluded in Winter 2016/2017; 38 students filled out this survey (*n* = 38).

The second cohort to take the JiTT and non-JiTT courses was the PharmD Class of 2021 (see Table 2 for the list of JiTT and non-JiTT courses offered for PharmD Class 2021 cohort of students). These students (*n* = 53) filled out the survey on perceptions about JiTT after finishing Integrated Microbiology & Virology class in Fall 2017. This cohort (*n* = 43) also filled out JiTT perception survey upon completion of the Integrated Immunology class during Winter 2017/2018.

Table 1 contains demographic information about the PharmD Classes of 2020 and 2021. Because the surveys were anonymous, it was impossible to discern which students opted not to participate. These statistics describe the totality of the respective cohorts.

### Survey Materials

At the end of the Fall 2016 iteration of the Integrated Microbiology & Virology course, students were provided a voluntary, anonymous survey containing 21 statements about the helpfulness of JiTT and the integrated case studies. All the 21 items listed as statements in the survey #1 are shown in Supplementary Table 1. The absence of JiTT literature in the health professions field made the use of an existing validated instrument ready for use difficult. Thus, the overall survey is a compilation of newly developed questions by the authors combined with questions modified from an existing survey on formative assessments in Biology education (33, 34). Cronbach-alpha was obtained to assess internal consistency and reliability. Students rated each statement on a 4-point scale ranging from *1 (I strongly disagree)* to *4 (I strongly agree)*. Each question had an option to indicate *I have no opinion*, which was treated as missing data. All items were positively phrased, e.g., “JiTT questions help me understand what it takes to be successful in this course.” The arithmetic mean of responses to these questions was computed for an aggregate measure of student perception of JiTT. Among these 21 statements, 13 statements asked students to evaluate the integrated case studies, such as “Integrated clinical cases helped me make connections across basic science and medicine”; the average score of these 13 items were averaged into a subscale of students' perception of the integrated case studies. The other eight questions asked about overall perception of JiTT. The average score of the entire survey had strong interrater reliability, Cronbach's Alpha 0.94. The survey had an open-ended question that asked how JiTT influenced students' learning of the course material.

Factor analysis on the first survey, for Fall 2016 Microbiology& Virology course, identified two distinct dimensions to our questions. Questions 1–13 were recognized as mostly belonging to one dimension, and these were the questions on case studies (explaining 47.55% of the variance). Questions 15–22 were recognized as mostly belonging to another dimension, and these were the questions about the overall perception of JiTT (explaining 24.37% of the variance). These two categories of questions cumulatively explained 71.92% of the variance. See Supplementary Table 3 for full results of factor analysis.

When the students were asked to rate their perception of JiTT in the Winter 2016/2017 iteration of the Immunology class, the survey was revised to (1) discard redundant questions, (2) increase the number of questions about different aspects of JiTT, (3) reduce the number of questions about the integrated case studies, (4) introduce several reverse-coded negatively phrased items as an attention check, and (5) be used in multiple courses that utilized JiTT pedagogy. The revised survey contained 22 items. All the 22 Items listed as statements in the revised survey #2 are shown in Supplementary Table 2. Five items were negatively-phrased, e.g., “JiTT questions made the course more difficult,” and 17 items were positively phrased, e.g., “JiTT provided structured opportunity for students to actively construct new knowledge of relevance to the lecture material.” Among these 22 items were two items that specifically asked students about integrated case studies: “JiTT case studies helped me reflect upon a topic that has already been covered in class,” and, “JiTT case studies helped me integrate basic science concepts with clinical case scenarios”; the average score on these two items created a subscale for students' perception of JiTT case studies.

For the revised survey, all items were rated on a 5-point scale in which *1* indicated “*Strongly disagree”* and *5* indicated “*Strongly agree*.” The survey eliminated the answer choice of “*I have no opinion,”* in order to compel respondents into providing feedback. All 22 items of this revised survey were utilized to compute an aggregate JiTT perception score. First, we computed the reverse score for the negatively-phrased items so that, for example, a score of 1 out of 5 was recoded as 5 out of 5. Scores were not transformed for the positively-phrased items. Then, an average score was computed using the scores for the positively-phrased items and the reversed scores for the negatively-phrased items such that higher scores reflect more-favorable perception of JiTT.

From this point forward, all surveys evaluating JiTT utilized the revised survey that was introduced to the Immunology class beginning with Winter 2016/2017. Factor analysis revealed that each iteration of this survey contained either three or four major categories of questions. However, in all instances, the top two categories which explained the most variance were perfectly mapped to the positively-phrased questions and negatively-phrased questions. For Winter 2016/2017 Immunology, the positively-phrased items explained 58.61% of the variance, and the negatively-phrased items explained 10.04% of the variance, with these top two dimensions cumulatively accounting for 68.65% of the variance. The survey for the Fall 2017 Microbiology & Virology class had 62.66% of the variance explained by the top two dimensions of questions: the positively-phrased items explained 50.26%, and the negatively-phrased questions explained an additional 12.40% of the variance. And, 73.86% of the variance was accounted for by the top two dimensions of the survey given to the Winter 2017/2018 Immunology class: 61.74% of the variance was accounted for by the positively-phrased items, and 12.12% of the variance was accounted for by the negatively-phrased items. See Supplementary Table 3 for full results of factor analysis.

In summary, factor analysis on the updated survey for Winter 2016-2017, Fall 2017, and Winter 2017-2018, found either three or four dimensions to the survey questions. In all three of these surveys, however, Dimensions 1 and 2 explained the most variance. In all instances, the “Dimension 1” questions perfectly mapped onto our positively-phrased questions, and the “Dimension 2” questions perfectly mapped onto our negatively-phrased questions. With the factor analysis confirming that the positively- and negatively-phrased questions achieved their intended effect, we believe it was appropriate to compute an aggregate JiTT score using all 22 items after reversing the scores for the negatively-phrased items. Interrater reliability of the aggregate JiTT score was very high; Cronbach's Alpha for Winter 2016/2017 Immunology, Fall 2017 Microbiology & Virology, and Winter 2017/2018 Immunology classes were, respectively, 0.96, 0.92, and 0.96. Therefore, comparisons about these three classes utilized the aggregate JiTT perception score as the dependent variable instead of the components of the scale (e.g., positively- or negatively-phrased items).

### Assessment of JiTT Pedagogy

Student's performance on knowledge-based questions in JiTT- vs. non-JiTT-based assessments were compared. Table 2 summarizes information about JiTT vs. non-JiTT courses, administered for PharmD Class 2020 and PharmD Class 2021 cohort of students, assessment data from which is included for comparison.

Three courses that did not rely on JiTT pedagogy approach are referred to as non-JiTT courses and these included the following: Pharmaceutical Biochemistry, offered in Fall Quarter; Foundations of Human Body & Disease I, II & III, offered in Fall, Winter and Spring Quarters, respectively; and Biotechnology, Pharmacogenomics and Precision Medicine, offered in Winter Quarter. While Pharmaceutical Biochemistry and Foundations of Human Body & Disease I, II & III were offered to Class 2020 and 2021 cohorts during their P1 Year of the curriculum, the Biotechnology, Pharmacogenomics and Precision Medicine course was offered until now only to Class 2020 cohort when they were enrolled into P3 Year. Class 2021 cohort is currently enrolled into Biotechnology, Pharmacogenomics and Precision Medicine course; hence, data presented for this course is only from Class 2020 cohort. Additionally, Biotechnology, Pharmacogenomics and Precision Medicine course is one course wherein a portion of the course had JiTT pedagogy implemented into it and another portion of the course that did not rely on JiTT pedagogy. The list of JiTT and non-JiTT courses that students from the two cohorts were enrolled into, is shown in Table 2.

The mean Kuder-Richardson Formula 20 (KR-20) values were used to assess consistent student performance on knowledge-based questions in individual assessments derived from JiTT vs. non-JiTT courses. We also compared the percentage of knowledge-based questions correctly answered for questions derived from JiTT-based vs. non-JiTT-based assessments. We compared two other objective measures, Discrimination Index (DISC) and Point Biserial (PB), for JiTT-based vs. non-JiTT-based assessments. DISC measures item quality and PB is a good discriminator between high-scoring and low-scoring students. Descriptive statistics for objective measures used for assessing knowledge-based learning outcomes are listed in Supplementary Table 4. Majority of the questions from JiTT or non-JiTT assessments were primarily knowledge-based. Every question in each assessment is mapped to one of the levels within Bloom's taxonomy. While all exams in JiTT vs. non-JiTT courses had knowledge-based questions, not all exams had higher order questions from Bloom's taxonomy included in them. A few exam questions were mapped to Bloom's taxonomy of “application” but these were not analyzed because they were very few of them. Therefore, we focused on knowledge-based questions.

### Data Analysis

SPSS Statistics for Windows, version 25 (SPSS Inc., Chicago, Ill., USA) was used for all data analysis. All analyses utilized two-tailed statistical significance at the *p* = 0.05 alpha level.

## Results

### Perception of JiTT

Demographic information presented in Table 1 suggests a diverse group of students from PharmD Classes of 2020 and 2021 that participated in the survey.

**Figure 2:**
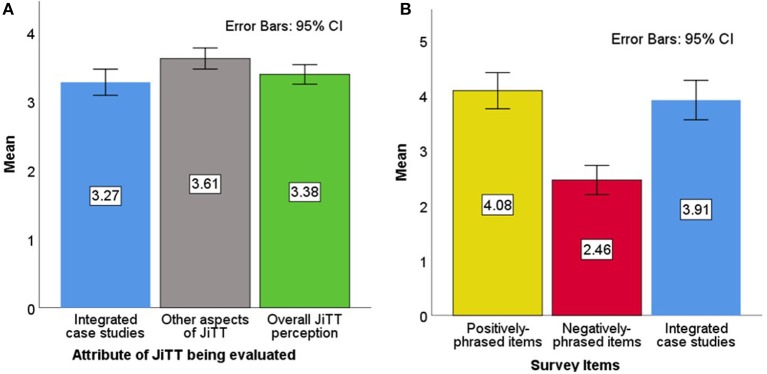
Class of 2020 perception of JiTT. Mean scores of three different attributes of JiTT evaluated for Fall 2016 Microbiology & Virology Class (**A**, left). Thirteen statements in the survey on integrated case studies were endorsed by students with an average score of 3.27 (*SD* = 0.62) out of a maximum of 4 points [blue bar]; eight positively-phrased items on other aspects of JiTT in the survey were endorsed by students with an average score of 3.61 (*SD* = 0.50) out of 4 points [gray bar]; all 21 items in the survey that reflected the overall JiTT perception score were endorsed by students with an average of 3.38 (*SD* = 0.46) out of 4 points [green bar]. Mean scores of items in JiTT survey evaluated for Winter 2016/2017 Immunology Class (**B**, right). Positively-phrased statements in the survey were endorsed by students with an average score of 4.08 (*SD* = 1.00) out of a maximum of 5 points [yellow bar]; negatively-phrased items in the survey were endorsed by students with an average score of 2.46 (*SD* = 0.80) out of 5 points [red bar]; integrated case studies were endorsed with an average of 3.91 (*SD* = 1.09) out of 5 points [blue bar].

The first JiTT survey was administered to first-year pharmacy students (Class of 2020) after the conclusion of the Integrated Microbiology & Virology class during Fall 2016. Items included in the first survey are listed in Supplementary Table 1. Thirteen items asked students for their endorsement (rated 1 through 4) of various positively-phrased statements specific to the integrated case studies that were utilized within the JiTT framework, and average endorsement was 3.27 (*SD* = 0.62) out of a maximum of 4 points; see Figure 2A. Eight items asked students for their endorsement on various statements related to the other aspects of JiTT, and average endorsement was 3.61 (*SD* = 0.50) out of 4 points. Scores on all 21 items of this survey were averaged into an overall JiTT perception score of 3.38 (*SD* = 0.46) out of 4 points. The Fall 2016 iteration of the Microbiology & Virology class was the only time when this version of the survey was used.

All subsequent coursework evaluated JiTT using the revised version of the survey in which possible scores ranged from *1* to *5*. Items included in the second revised survey are listed in Supplementary Table 2. The dependent variable for this survey was the aggregate JiTT perception score, i.e., the arithmetic mean of the positively-phrased items and the reversed scores of the negatively-phrased items. The Class of 2020 filled out this survey after the Winter 2016/2017 Immunology class, and aggregate JiTT perception was 3.96 out of 5 (*SD* = 0.87). Endorsement of the positively-phrased items had an average score of 4.08 (*SD* = 1.00) out of a maximum of 5 points; see Figure 2B. For the negatively-phrased items, the average raw endorsement was at 2.46 (*SD* = 0.80) out of 5 points; the relatively low score indicates that students typically disagreed with the statements that found faults with JiTT. For the subset of questions that asked students to evaluate the case studies, the average score was 3.91 (*SD* = 1.09) out of 5 points.

**Figure 3:**
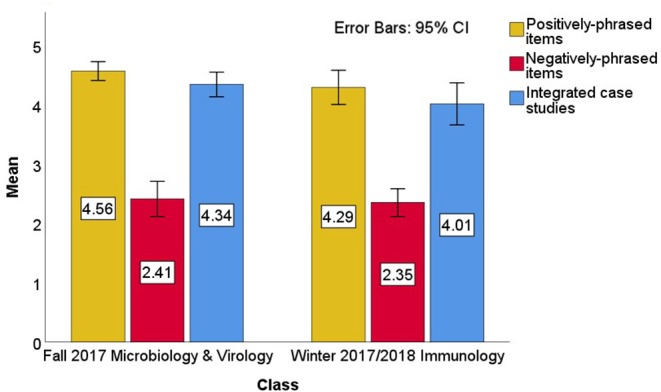
Class of 2021 Perception of JiTT. Mean scores of items in JiTT survey evaluated for Fall 2017 Microbiology & Virology Class. Positively-phrased statements in the survey were endorsed by students with an average score of 4.56 (*SD* = 0.57) out of a maximum of 5 points [yellow bar]; negatively-phrased items in the survey were endorsed by students with an average score of 2.41 (*SD* = 1.07) out of 5 points [red bar]; integrated case studies were endorsed with an average of 4.34 (*SD* = 0.75) out of 5 points [blue bar]. Mean scores of items in JiTT survey evaluated for Winter 2017/2018 Immunology Class. Positively-phrased statements in the survey were endorsed by students with an average score of 4.29 (*SD* = 0.94) out of a maximum of 5 points [yellow bar]; negatively-phrased items in the survey were endorsed by students with an average score of 2.35 (*SD* = 0.76) out of 5 points [red bar]; integrated case studies were endorsed with an average of 4.01 (*SD* = 1.15) out of 5 points [blue bar].

The next cohort to experience these two JiTT classes was the Class of 2021. These students took the Microbiology & Virology course during Fall 2017. The aggregate JiTT perception was 4.34 out of 5 (*SD* = 0.58). Average endorsement of the positively-phrased items was quite high, 4.56 (*SD* = 0.57) out of 5 points; see Figure 3. Raw endorsement for the negatively-phrased items had an average of 2.41 (*SD* = 1.07) out of 5 points. And, students found the case studies quite helpful, with an average score of 4.34 (*SD* = 0.75) out of 5 points on the items asking about JiTT case studies.

The final class in this study was the Winter 2017/2018 Immunology class, which had an aggregate JiTT perception score of 4.14 (*SD* = 0.84). Student endorsement of the positively-phrased survey items was 4.29 (*SD* = 0.94) out of 5 points, and their raw endorsement of the negatively-phrased items had an average of 2.35 (*SD* = 0.76) out of 5 points; see Figure 3. The average score on the questions asking about case studies was 4.01 (*SD* = 1.15) out of 5 points.

**Figure 4:**
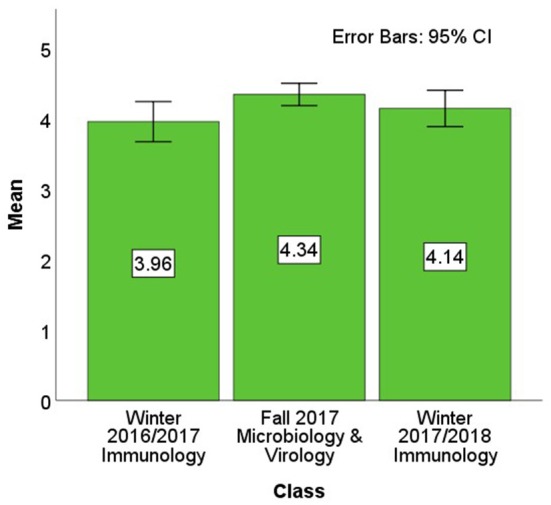
Aggregate perception of JiTT. Aggregate mean overall JiTT perception score was calculated from the mean values derived from the positively-phrased items and the reversed responses from the negatively-phrased items. The aggregate JiTT perception score for the Winter 2016/2017 Immunology class of 2020 cohort was 3.96 (*SD* = 0.87) out of 5, for the Fall 2017 Microbiology & Virology class of 2021 cohort was 4.34 (*SD* = 0.58) out of 5, and for the Winter 2017/2018 Immunology class of 2021 cohort was 4.14 (*SD* = 0.84) out of 5.

The aggregate JiTT perception scores for the Winter 2016/2017 Immunology class, the Fall 2017 Microbiology & Virology class, and the Winter 2017/2018 Immunology class were compared in an analysis of variance (ANOVA) model (see Figure 4 for the scores being compared). This ANOVA analysis omitted the survey from the Fall 2016 survey of the “Integrated Microbiology & Virology” class because its responses were on a four-point scale whereas the latter three surveys were on a five-point scale and the two surveys did not use the same items. Results from the ANOVA model on aggregate JiTT perception suggest that JiTT was comparably well-received across these classes, *F*(2, 131) = 2.94, *p* = 0.057, *R*^2^ = 0.43. A Tukey's HSD *post-hoc* test found that the Winter 2016/2017 Immunology class was less-favorably rated than the Fall 2017 Microbiology & Virology class, *p* = 0.046 All other pairwise comparisons were not statistically significant, all *p*-values > 0.4.

Despite statistical significance in *post-hoc* testing, however, the scores were quite high overall and the ANOVA accounted for very little variance, and so a larger picture of students' perception of JiTT was warranted by combining all the JiTT perception ratings. After aggregating the scores from the Fall 2017 Microbiology & Virology course and both instances of the Immunology course, students' grand mean JiTT perception score was quite favorable, 4.17 (*SD* = 0.77) out of 5.

### Objective Comparisons Between JiTT vs. Non-JiTT Assessments

**Figure 5:**
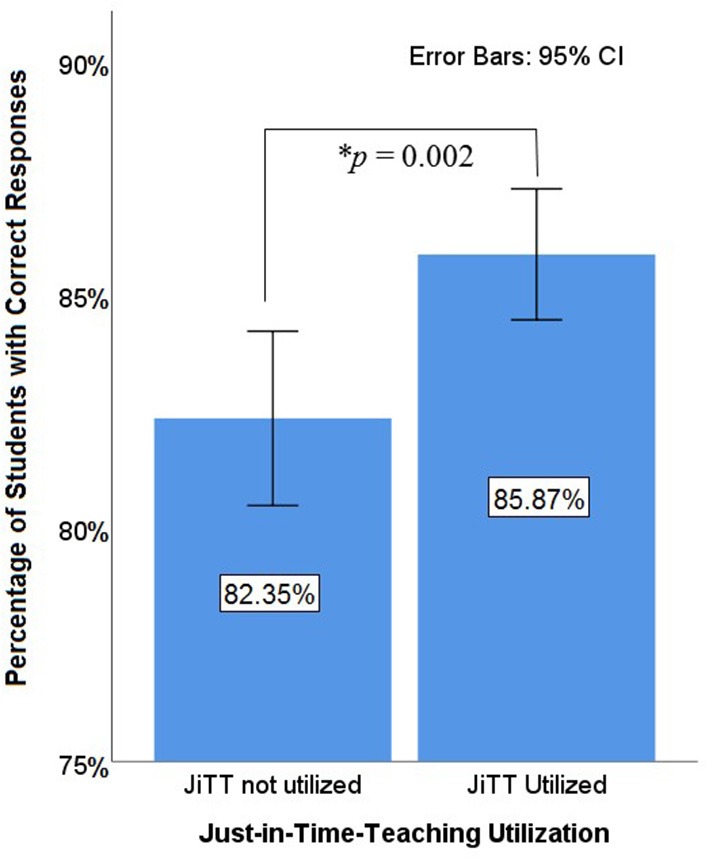
Student performance on individual exam items. Student learning outcomes were assessed by the average performance on individual test questions. Student's performance was compared on individual exam questions that asked about topics taught with JiTT and topics without JiTT. Knowledge-based questions were only analyzed. Each exam question was one observation. For questions on topics that utilized JiTT, 85.87% of students got the questions correct. For questions on topics not utilizing JiTT, 82.35% of the students got the questions correct. The difference is statistically significant, *t*_(682)_ = 3.05, *p* = 0.002.

**Figure 6:**
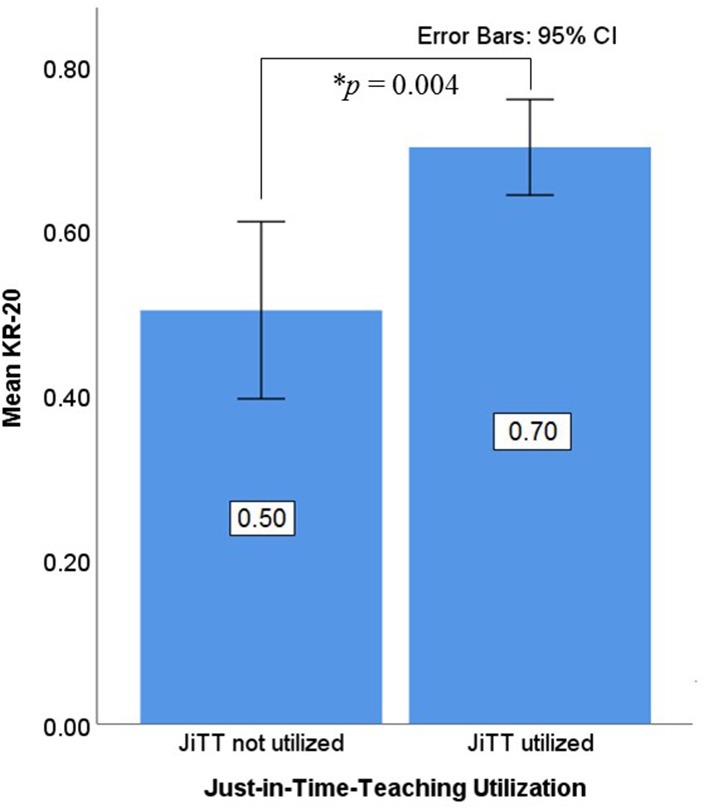
Exam-Level Consistency. The Kuder-Richardson Formula 20 (KR-20) values were used to assess consistent student performance in JiTT vs. non-JiTT courses. Mean KR-20 values for knowledge-based questions were extracted from individual assessments pertaining to JiTT and non-JiTT courses. There were 15 exams on topics that were not taught with JiTT (JiTT not utilized). With each exam as one observation, the average KR-20 score was 0.50, *SD* = 0.21. There were 14 exams on topics taught with JiTT (JiTT utilized). With each exam as one observation, the average KR-20 score was 0.70, *SD* = 0.11. This difference between JiTT vs. non-JiTT exams is statistically significant, *t*_(27)_ = 3.17, *p* = 0.004.

We conducted analyses on whether objective measures would have significant differences based on whether or not JiTT was utilized in teaching the material. This data presented in Figures 5, 6 reflects consistency and learning outcomes in knowledge-based questions. Table 2 has a list of JiTT and non-JiTT courses, administered for PharmD Class 2020 and PharmD Class 2021 cohort of students, assessment data from which was used for comparison. One analysis focused on objective student performance, which we defined as the percentage of students who correctly answered each knowledge-based question on an exam. Each unit of observation was one knowledge-based question from an exam, and the sample was every exam from a set of courses that utilized JiTT (Fall 2016 Microbiology & Virology, Winter 2016/2017 Immunology, Fall 2017 Microbiology & Virology, and Winter 2017/2018 Immunology, and Winter 2018/2019 Biotechnology, Pharmacogenomics & Precision Medicine) and a set of courses—taken by the same students—that did not utilize JiTT (Fall 2016 and Fall 2017 Pharmaceutical Biochemistry, Fall 2016 and Fall 2017 Foundations of Human Body & Disease I, Winter 2016/2017 and Winter 2017/2018 Foundations of Human Body & Disease II, Spring 2017 and Spring 2018 Foundations of Human Body & Disease III, and Winter 2018/2019 Biotechnology, Pharmacogenomics & Precision Medicine). From these classes, 684 distinct knowledge-based questions were identified.

On average, exam questions from courses that utilized JiTT were answered correctly by 85.87% of the students (*SD* = 14.37%). Exam questions from classes that did not utilize JiTT had an average of 82.35% performance (*SD* = 15.20%). This difference was statistically significant, *t*_(682)_ = 3.05, *p* = 0.002, which suggests JiTT is helpful for student learning (Figure 5). See Supplementary Table 4 for item statistics data.

KR-20 scores were analyzed as another objective measure. Because KR-20 scores apply to the consistency of an entire exam, the unit of observation in this analysis was each distinct exam. From the courses listed above, 29 distinct exams were identified: 15 exams on topics that were not taught with JiTT and 14 exams on topics taught with JiTT. For courses that utilized JiTT, the mean KR-20 score for exams was 0.70, *SD* = 0.11. Exams from courses that did not utilize JiTT had mean KR-20 score of 0.50, *SD* = 0.21. The difference was statistically significant, *t*_(27)_ = 3.17, *p* = 0.004, indicating that JiTT-based courses had more-consistent exams (Figure 6). See Supplementary Table 4 for item statistics data. The other two objective measures analyzed, Discrimination Index (DISC that measures item quality) and Point Biserial (PB, a good discriminator between high-scoring and low-scoring students) were not statistically significant between JiTT-based vs. non-JiTT-based courses, *t*_(682)_ = 0.306, *p* = 0.759 and *t*_(682)_ = 0.825, *p* = 0.410, respectively (see Supplementary Table 4 for item statistics data). This suggests items from all the assessments were equally reliable.

Survey analysis showed that students perceived JiTT was beneficial to their active learning of the course material and helped them keep track of the course content. Students' performance data comparing JiTT- vs. non-JiTT- based courses indicated that JiTT was beneficial for student learning. JiTT pedagogy was conducive for enhancing knowledge-based skills and this is based on assessment of student learning outcomes in JiTT-based courses vs. non-JiTT-based courses.

## Discussion

JiTT is an active learning pedagogy that was successfully implemented across various scientific disciplines (1, 4). However, the usage of JiTT has not been reported in a PharmD curriculum. Our goal toward implementation of JiTT as a meaningful learning tool was to enhance conceptual knowledge of core topics within Integrated Microbiology & Virology and Integrated Immunology courses. The idea of implementing JiTT active learning technique in a flipped classroom model is to divert students from sheer memorization of the required course material prior to any major assessments. Hence, JiTT active learning pedagogy was implemented for both courses that are part of the Biomedical Sciences curriculum offered to PharmD students during the first year of their program (31, 32). Preparation of JiTT assignments and case studies were an integral part of PharmD Integrated Microbiology & Virology and Integrated Immunology courses.

Results from both the surveys demonstrated that the overall perception of JiTT in PharmD Integrated Microbiology & Virology and Integrated Immunology curricula offered during Fall Quarter 2016 through Winter Quarter 2018 was favorable. The aggregate mean score for overall perception of JiTT, from survey analysis of JiTT implemented in two courses for two different cohorts of students, was quite high, indicating the positive influence of JiTT on students' learning of the course materials. These observations are in agreement with the previously reported student's perception of JiTT-based teaching approach for an undergraduate-level Immunology course (19, 20). Responses to an open-ended query on how JiTT influenced learning of the course material indicated that students perceived integrated case studies administered in JiTT format to be thought-provoking that helped identify their areas of improvement in certain areas of basic sciences. Students also felt participation in JiTT assignments markedly improved their understanding of the relevant course topics, helped participate in discussions involving case studies, be on track with the course material while helping them prepare for exams and retain information better. This is consistent with what was observed earlier that JiTT augmented learning of key points, increased learner participation, and enhanced learner retention of core concepts (7, 19, 20). Student-centered active learning pedagogies implemented into integrated basic science curricula facilitate student engagement during in-class discussions and help students understand, retain and apply basic science concepts to clinical practice (19–24).

To summarize students' responses from positively-phrased items in the survey, JiTT was beneficial, helped students enhance their knowledge-based skills and JiTT created an interactive active learning environment. This aligns with what was reported earlier that significantly favorable perception of JiTT may have been because JiTT educational experience matched with the evolving needs of millennial learners, which included: interactive learning, self-directed teaching and use of novel digital teaching technological methods (8, 21). Statistical analysis showed significantly favorable overall perception of JiTT when Integrated Immunology courses were compared. Our data is in agreement with reported literature that JiTT serves as an effective learning tool that helps novice learners to recognize, understand, and retain the jargon before engaging in deeper learning of Immunology concepts including integrated case studies (10, 19, 20). The consistently low scores for the negatively-phrased items indicate that students disagreed with disfavorable statements about JiTT instructional pedagogy.

Comparison of student performance on knowledge-based questions between JiTT vs. non-JiTT courses from major assessments indicated that JiTT was helpful in student's learning of knowledge-based concepts. This is in agreement with previously reported observation that JiTT methodology effectively enhanced knowledge-based skills required for understanding of core health-care professional curricular topics (2, 6–10, 15). Additionally, analysis of mean KR-20 values from each assessment also showed that courses with JiTT pedagogy had consistent exam performance compared to non-JiTT courses offered to the same cohorts. This data suggests that teaching a concept with JiTT is correlated with better outcomes and more-consistent exams when compared to non-JiTT approaches. The current data is a promising initial step in validating the usefulness of JiTT in a pharmacy program.

One limitation of the study was the usage of anonymous surveys. The rationale behind anonymity was to provide students the comfort and freedom to express their opinion of the quality of teaching. Without any ability to link the students to their responses, all observations were treated as independent in the analyses, and a time-series analysis was impossible. Another limitation of the study was the usage of two different surveys. The Microbiology & Virology course made extensive use of integrated case studies, and the Fall 2016 iteration of the class was the first time this class was offered at this particular university. Therefore, the JiTT survey was catered to that particular course, and many items focused on the integrated case studies. When the time came to assess students' perception of JiTT in the next course, a survey was created that could be used for any course that utilized JiTT pedagogy. Because data was collected after an academic quarter of applied use of JiTT, these findings should reflect valid student perceptions. Another limitation was that JiTT utilization was confounded by instructors and by courses—each course only had one instructor, JiTT was utilized in certain courses but not others, and it is possible that the courses varied in difficulty, thus necessitating the analyses on item reliability. Although a fully-factorial design would eliminate this confounder, doing so was impossible due to the limited number of faculty assigned to courses at the time the courses were taught.

Current study demonstrated that JiTT was advantageous to students in that it compelled students to read and be better prepared for the course material posted online for an upcoming lecture topic. In agreement with Novak et al. JiTT helped course instructors adapt to student's learning needs (1). Course instructors waded into the task of tailoring and fine tuning each class session, based on learning gaps identified *via* student responses to JiTT assignments, instead of taking the traditional approach of one size fits all. Consistent with previous observation on usefulness of JiTT in an undergraduate Immunology course (19, 20), it was also observed during class sessions that students demonstrated competency with JiT learning skills through increased student participation and greater student engagement. Our results suggest that JiTT assessments were linked with higher student performance and consistency.

## Conclusions

Based on results from both the surveys, students perceived JiTT was beneficial to their active learning of the course material and helped them keep track of the course content. Students' performance data comparing JiTT- vs. non-JiTT- based courses indicated that JiTT was helpful for student learning and JiTT pedagogy was conducive for enhancing knowledge-based skills. The current data is a promising step in validating the usefulness of JiTT in a pharmacy program and lays the foundation for a direct comparison between a traditional lecture style and JiTT active learning pedagogy implemented into PharmD curricula.

## Data Availability Statement

The datasets generated for this study are available on request to the corresponding author.

## Ethics Statement

The studies involving human participants were reviewed and approved by Institutional Review Board of Marshall B. Ketchum University. Written informed consent for participation was not required for this study in accordance with the national legislation and the institutional requirements.

## Author Contributions

CM developed JiTT pedagogy for the two courses, implemented JiTT while being a Course Coordinator and/or Course Instructor for three courses, participated in writing and facilitating case studies, participated in JiTT survey design, and wrote the manuscript. ET-C implemented JiTT while being a Course Coordinator and/or Course Instructor for three courses, participated in writing and facilitating case studies, participated in JiTT survey design and wrote the manuscript. HH participated in JiTT survey design, created revised version of the JiTT survey, performed data analysis, and wrote the manuscript. JS was the Course Coordinator for a non-JiTT Pharmaceutical Biochemistry course and contributed to writing the manuscript. AN was the Course Coordinator for a non-JiTT Foundations of Human Body & Disease I, II, and III course series and contributed to writing the manuscript. DB originally implemented JiTT for an undergraduate Immunology course, participated in survey design, and contributed to writing the manuscript.

### Conflict of Interest

The authors declare that the research was conducted in the absence of any commercial or financial relationships that could be construed as a potential conflict of interest. The handling editor declared a shared collaboration with one of DB, and was assigned to this manuscript at the request of the Frontiers Editorial Office.
