# Supplementary material for: Analysis of Student Perceptions of Just-In-Time Teaching Pedagogy in PharmD Microbiology and Immunology Courses
Source: Front Immunol. 2020 Feb 28;11:351. doi: 10.3389/fimmu.2020.00351 (PMC7058994; doi:10.3389/fimmu.2020.00351)
Supplement: Supplementary file 1 [file Table_1.DOCX]

Supplementary Material

# SUPPLEMENTARY TABLES

**Supplementary Table 1.** List of Items for Survey #1.

| Item No. | Item statement |
| --- | --- |
| 1 | Integrated clinical cases helped me to understand basic microbiology and virology principles |
| 2 | Integrated clinical cases helped me to understand on how to apply what I have learned |
| 3 | Integrated clinical cases offered me with a more extended view of the topics I learnt |
| 4 | Integrated clinical cases stimulated interest and questions that anticipate future learning |
| 5 | Integrated clinical cases helped me make connections across basic science and medicine |
| 6 | Integrated clinical cases helped me develop skills in learning problem-solving and teamwork |
| 7 | Integrated clinical cases helped me apply, build upon and integrate knowledge across courses |
| 8 | Integrated clinical cases helped me develop my skills in communicating the clinical findings |
| 9 | Integrated clinical cases enabled me to achieve a deeper understanding of the basic science principles relevant to the clinical cases reviewed |
| 10 | Integrated cases improved my ability to evaluate and manage patient cases |
| 11 | Experiences like this would enhance my motivation for exploring basic science principles relevant to clinical problems |
| 12 | Understanding basic science principles relevant to clinical problems would contribute to better patient care |
| 13 | I would recommend integrated clinical cases teaching in other basic sciences courses |
| 14 | The use of JiTT questions influenced my learning in this course |
| 15 | Overall, JITT questions are beneficial to me |
| 16 | Overall, JiTT questions are beneficial to the instructor |
| 17 | JiTT questions help me identify what material I am expected to learn in this course |
| 18 | JiTT questions help me understand what it takes to be successful in this course |
| 19 | JiTT questions gave me feedback on what I still need to learn in this course |
| 20 | JiTT questions help me take control of my own learning in this course |
| 21 | Discussing JiTT questions with other students helped me learn |
| 22 | How the use of JiTT questions influenced your learning in this course? [free response, open-ended question] |

**Supplementary Table 2.** List of Items for Revised Survey #2.

| Item No. | Item statement |
| --- | --- |
| 1 | JiTT questions helped me keep track of the material taught in class |
| 2 | JiTT questions enhanced active learning |
| 3 | JiTT assignments and case studies gave me extra work |
| 4 | JiTT made the material presented in lecture easier to understand |
| 5 | The use of JiTT questions influenced my learning in this course |
| 6 | Overall, JITT questions are beneficial to me |
| 7 | JiTT questions help me identify what material I am expected to learn in this course |
| 8 | JiTT questions help me understand what it takes to be successful in this course |
| 9 | JiTT questions give me feedback on what I still need to learn in this course |
| 10 | JiTT questions help me take control of my own learning in this course |
| 11 | JiTT had no bearing on how well I learned the material |
| 12 | JiTT questions made the course more difficult |
| 13 | JiTT made it difficult to understand how to get a good grade in this course |
| 14 | JiTT assignments helped me stay prepared and be focused before each class session |
| 15 | JiTT provided structured opportunity for students to actively construct new knowledge of relevance to the lecture material |
| 16 | JiTT assignments warm up students to think about the upcoming lecture material making it easier to understand |
| 17 | JiTT case studies helped me reflect upon a topic that has already been covered in class |
| 18 | JiTT created an opportunity for an interactive, active learning, focused class environment |
| 19 | JiTT case studies helped me integrate basic science concepts with clinical case scenarios |
| 20 | JiTT helped me link the work-at-home experience to classroom experience |
| 21 | JiTT helped stay caught-up with the course material instead of falling behind |
| 22 | JiTT increased student workload |

**Supplementary Table 3.** Factor Analysis of JiTT Perception Surveys

| Version of Survey | Class | Percentage of Survey Variance Explained | | | |
| --- | --- | --- | --- | --- | --- |
|  |  | Dimension 1 | Dimension 2 | Dimension 3 | Dimension 4 |
| 1 | Fall 2016 Microbiology & Virology | 47.55 | 24.37 | - | - |
| Revised Survey Created | | | | | |
| 2 | Winter 2016-2017 Immunology | 58.61 | 10.04 | 6.01 | 5.58 |
|  | Fall 2017 Microbiology & Virology | 50.26 | 12.40 | 5.80 | 4.94 |
|  | Winter 2017-2018 Immunology | 61.74 | 12.12 | 5.44 | - |
| *Note:* Only the dimensions with statistically-significant additional accounting for the variance are listed. | | | | | |

**Supplementary Table 4.** Descriptive Statistics for Objective Measures

| Unit of Measurement | Measure | JiTT Utilized | | JiTT Not Utilized | | *p*-value |
| --- | --- | --- | --- | --- | --- | --- |
|  |  | Mean (SD) | N | Mean (SD) | N |  |
| Exam | KR-20 | 0.70 (0.11) | 14 | 0.50 (0.21) | 15 | 0.004 |
| Exam Question | Proportion of Students with Correct Responses | 0.86 (0.14) | 265 | 0.82 (0.15) | 419 | 0.002 |
|  | DISC | 0.21 (0.17) | 265 | 0.22 (0.17) | 419 | 0.759 |
|  | Point Biserial | 0.29 (0.19) | 265 | 0.28 (0.19) | 419 | 0.410 |
| *Note:* *p*-values are for unpaired Student’s *t*-tests comparing items or exams that utilized JiTT with those that did not utilize JiTT | | | | | | |
